# The early days of late blight

**DOI:** 10.7554/eLife.00954

**Published:** 2013-06-18

**Authors:** Paul RJ Birch, David EL Cooke

**Affiliations:** 1 **Paul RJ Birch** is at the Division of Plant Sciences, University of Dundee, Dundee, United Kingdom and the James Hutton Institute, Dundee, United Kingdompaul.birch@hutton.ac.uk; 2 **David EL Cooke** is at the James Hutton Institute, Dundee, United Kingdomdavid.cooke@hutton.ac.uk

**Keywords:** Phytophthora infestans, Solanum tuberosum, Herbarium, ancient DNA, Other

## Abstract

Large-scale DNA sequencing of samples of foliage collected in the 19th century from plants infected with late blight has shown that the potato famines of the 1840s were triggered by a single clonal lineage of *Phytophthora infestans*, called HERB-1, which persisted for at least 50 years.

**Related research article** Yoshida K, Schuenemann VJ, Cano LM, Pais M, Mishra B, Sharma R, Lanz C, Martin FN, Kamoun S, Krause J, Thines M, Weigel D, Burbano HA. 2013. The rise and fall of the *Phytophthora Infestans* lineage that triggered the Irish potato famine. *eLife* **2**:e00731. doi: 10.7554/eLife.00731

**Image** The pathogen *P. infestans* has spread around the world from Mexico since the 1840s

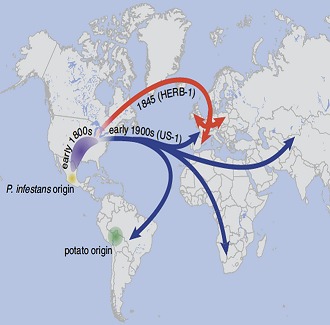

There can be few diseases of plants that have had a more profound effect on human health, society and politics than potato late blight. Emerging in the mid-1840s, the disease rapidly became pandemic, precipitating the Great Famine, which led to almost a million deaths in Ireland and the emigration of many more, plus considerable loss of life and extreme political upheaval throughout mainland Europe (Zadoks, 2008).

The water mould *Phytophthora infestans* was first identified as the probable cause of late blight by Miles Joseph Berkeley in 1846, almost 20 years before Louis Pasteur formally proposed the germ theory of disease. As such, it has been credited with establishing the field of plant pathology, and is a major milestone in the foundation of epidemiology as a scientific discipline. Now, more than 160 years later, Hernán Burbano of the Max Planck Institute for Developmental Biology and co-workers have combined the power of next-generation sequencing technology with the availability of herbarium samples to peek through a 50-year window of history at the genome of the pathogen that contributed to so much human suffering (Yoshida et al., 2013).

Work in the 1990s by researchers at Cornell University revealed a dominant lineage of *P. infestans*, dubbed US-1, which was identified in 13 countries on four continents. This lineage was defined by nuclear DNA markers and, crucially, a mitochondrial haplotype termed Ib, and the Cornell team proposed that it had emerged from Mexico in the 1840s to cause severe crop losses on a global scale (Goodwin et al., 1994). However, this idea was challenged when researchers at North Carolina State University identified a different type of mitochondrial DNA in herbarium samples of potato late blight collected between 1845 and 1886 (Ristaino et al., 2001).

Now, Burbano and co-workers—including Kentaro Yoshida of the Sainsbury Laboratory and Verena Schuenemann of the University of Tübingen as joint first authors, plus colleagues at other centres in Germany and the US—have sequenced the genome of *P. infestans* from 11 infected leaf samples that were gathered in Ireland, Great Britain, Germany and Canada between 1845 and 1896 (Yoshida et al., 2013). They found that the pathogen strains in all samples were representatives of a single lineage, which they have called HERB-1. This strain of *P. infestans* was responsible for the first global migration of the pathogen in the 1840s, and then persisted for at least 50 years. It has not been observed in modern times, but is clearly a close relative of US-1. Analysis of both mitochondrial and nuclear DNA showed HERB-1 and US-1 to be sister groups in a clade distinct from contemporary genotypes.

Mexico is the foremost centre of *P. infestans* diversity in the world, but Yoshida, Schuenemann et al. propose that HERB-1 and US-1 emerged from a metapopulation that was established outside of Mexico in the early 19^th^ century and that US-1 displaced HERB-1 at some point in the 20^th^ century (Figure 1). The new work also emphasises the threats associated with the movement of pathogens between continents, and highlights the occurrence of waves of pathogen migration and genotype displacement. Indeed, dramatic shifts in *P. infestans* populations are still being observed today with, for example, the aggressive genotype 13_A2 displacing other genotypes across Europe over a period of less than three years (Cooke et al., 2012), and the rapid emergence of genotype US-22 in the eastern USA in 2009 (Fry et al., 2013). Moreover, it should be possible to use the newly obtained DNA sequences to develop markers that allow 20^th^ century samples to be tested for the presence of HERB-1. It will be interesting to figure out what happened between 1900 and the 1970s, when new populations began to displace US-1 in Europe (Figure 1).

**Figure 1.**
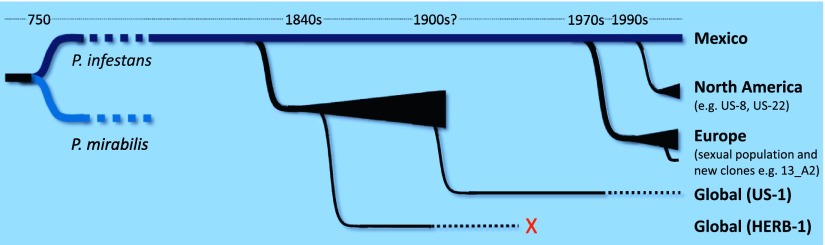
Schematic representation of the time-line for the emergence of major lineages of *Phytophthora infestans,* the pathogen that causes late blight in potatoes, as proposed by Burbano and co-workers (Yoshida et al., 2013). *P. infestans* and *P. mirabilis* (a pathogen that does not infect potatoes) are thought to have diverged from a common ancestor about 1200 years ago. Since then there have been at least three major migrations of *P. infestans* from a sexually reproducing population in Mexico (shown by black triangles). The first involves a metapopulation established in an unknown region, from which HERB-1 and US-1 emerged and spread around the world. US-1 is rarely found in contemporary populations (as indicated by the dotted black line) and HERB-1 is not found anywhere today (red cross). A shipment of potatoes from Mexico into Europe during a drought in 1976 is thought to be the source of the population of *P. infestans* that displaced US-1 in Europe and resulted in the emergence of aggressive new clones such as 13_A2 (also known as Blue13). Lastly, independent migrations into the US resulted in the dominance of the US-8 lineage, although this is now being displaced by other lineages such as US-22. The precise timing of the displacement of HERB-1 by US-1 remains undefined.

Although these latest results provide new insights into the pathogen responsible for famines in the 19^th^ century, questions remain about the precise cause and timing of subsequent waves of new lineages. Yoshida et al. found no evidence for the positive selection of particular genes that might have driven displacement of HERB-1 by US-1. They hypothesised that cross breeding commercial potatoes with *Solanum demissum*, a wild species of potato that is resistant to some lineages of *P. infestans*, in Europe in the 1930s-1950s may have driven selection against HERB-1 and, by inference, favoured the US-1 strain that displaced it. This is an interesting theory, and there is a clear example of a widely grown commercial cultivar (the Pentland Dell) that contained resistance genes from *S. demissum* that were subsequently ‘broken’ by a changing pathogen population (Malcolmson, 1969; Fry, 2008). However, more data are needed to test this hypothesis.

Yoshida et al. propose that US-1 and HERB-1 come from a single metapopulation that was itself derived from Mexican populations that were likely to have been exposed to *S. demissum*. Should we expect to see a difference in the response of the different lineages of *P. infestans* to this form of resistance? Understanding the co-evolution of hundreds of virulence genes in the pathogen (Haas et al., 2009) with potentially similar numbers of resistance genes in potato (Jupe et al., 2012) is challenging, and datasets such as this should help to answer this question.

HERB-1 existed as a clonal (asexual) lineage for at least 50 years with a generation time as short as a few days during each cropping season. The long-term evolutionary consequences of such clonality are that deleterious mutations can accumulate. The decline in fitness caused by these mutations is an example of Muller’s ratchet in action (Goodwin, 1997) and might explain why lineages emerging from the sexually reproducing metapopulation described by Yoshida et al. displaced HERB-1. Yoshida et al. provide further support for such a scenario by showing that HERB-1 was diploid but US-1 and contemporary clonal lineages often have increased ploidy. It could be that such increased genetic diversity, providing a buffering capacity against deleterious mutations, offered subsequent lineages an evolutionary advantage over HERB-1.

Emerging diseases present serious threats to human health and to the health of livestock, crops and natural ecosystems. We have seen an increase in emerging diseases in the past two decades, likely due to increased trade, transport and travel, and perhaps due also to changes in climate (Fisher et al., 2012). By providing significant insights into the make-up of one of the most notorious emerging diseases in history, the work of Burbano, Yoshida, Schuenemann and colleagues offers us a valuable opportunity to learn from the past.
